# Reply to Comment on “Enumeration of *Escherichia coli* in Probiotic Products. *Microorganisms* 2019, 7, 437”

**DOI:** 10.3390/microorganisms8020242

**Published:** 2020-02-12

**Authors:** Camille Zimmer, Caetano C. Dorea

**Affiliations:** Department of Civil Engineering, University of Victoria, Victoria, BC V8P 5C2, Canada; caetanodorea@uvic.ca

We thank Wassenaar and colleagues for their Comment on our recent paper [1] and appreciate the opportunity to address it. Although not entirely explicit, their Comment seems to suggest that our assessment of *E. coli* in two probiotic products is possibly underestimated due to the enumeration technique we adopted.

In our work we utilized the Colilert Quanti-tray/2000 system [2] which is a well-known method for *E. coli* enumeration based on Most Probable Number (MPN). There is a plethora of published literature reporting on utilization of this technique to enumerate *E. coli* in a variety of matrices and in a wide range of concentrations. This is in stark contrast with their Comment that seems to imply that the applicability of such MPN technique is limited to surface water samples with low coliform concentrations.

We appreciate the underpinning differences between the enumeration technique chosen for our study and those recognized under the regulatory requirements of probiotic manufacturing. We are open to accept that there may be differences in results within and between these varied techniques. Our estimates of *E. coli* in the two sampled probiotics can be interpreted considering such a possibility, until such a comparison between methods (which was beyond the scope of our study) is done for these probiotics. For now, to our knowledge, they are the only published results that have been subject to peer review.
